# Supplementary material for: Adaptation supports short-term memory in a visual change detection task
Source: PLoS Comput Biol. 2021 Sep 17;17(9):e1009246. doi: 10.1371/journal.pcbi.1009246 (PMC8480767; doi:10.1371/journal.pcbi.1009246)
Supplement: Attachment — Submitted filename: STSPNet_rebuttal.pdf [file pcbi.1009246.s010.pdf]

Dear Editors,

Please find the letter with the detailed responses to the reviewers appended. In it we include the comments from the reviewers in blue.

[1] A letter containing a detailed list of your responses to the review comments and a description of the changes you have made in the manuscript. Please note while forming your response, if your article is accepted, you may have the opportunity to make the peer review history publicly available. The record will include editor decision letters (with reviews) and your responses to reviewer comments. If eligible, we will contact you to opt in or out.

Comments to the Authors:

<u>Reviewer #1</u>: In this paper the authors compare two neural network models with neural data obtained from a visual cortical area as mice learn to solve a task. In particular, the authors implement two models which utilise different mechanisms underlying short-term memory: the STPNet using short-term synaptic depression and an RNN model using persistent activity through recurrent weights. These models were compared with neural and behavioural data taken from a mouse performing a visual change detection task. The results seem to suggest that the STPNet model better captures the neuronal data. This combination of data and model is novel and is of potential interest to not just computational neuroscience but also the wider neuroscience community. The paper is well written, but there is a lack of clarity in several places. In addition, at the present there are substantial issues with the paper that should be addressed.

Major points

1. The authors pretrained a CNN which was used to generate low-dimensional features to both models considered. CNNs are often interpreted as models of the visual cortex (e.g. work from the Dicarlo lab). This means that the connections within the CNN model should themselves have STP. Similarly, a recurrent CNN (e.g. Nayebi et al. NeurIPS 2018) could in principle be studied. These two models could then be compared with one another. One option would be to adapt the CIFAR dataset to directly train the task that mice were trained on, potentially augmenting the dataset used for animal training with a larger dataset. We understand that this might complicate things, but it should be at least discussed in detail and these two components of the model better explained (see also major point 3 below).

The roles of short-term plasticity and recurrent connections in the feature preprocessing stage are interesting topics. We have now included a section in the Discussion describing this (**“Scope and limitations of the model”, line 311**). However, since this preprocessing is not the main focus of our paper we only applied adaptation or recurrence at the feature embedding level. Since we have adaptation at the input of the network we are studying, we do not believe that having one or multiple stages of adaptation in the preprocessing stage will affect our results.

2. In the STPnet the STD parameters are fixed (apart from W which are learnt via backprop). Whereas in the RNN model all parameters are learnt via backprop. This makes the comparison somewhat unfair as the dynamic component of the RNN (i.e. the recurrent weights) are modified through backprop but the equivalent component in the STPnet is not. What would happen if the recurrent weights in the RNN are fixed to values equivalent to the STD dynamics (e.g. w_rec ~ 0.5)? Conversely what happens if you also train the STD parameters using backprop? There is wide evidence to support that STP is plastic in itself (see for example a model that captures this interaction Costa et al. 2015 eLife). In general, the current RNN model does not seem to have any decay, which is odd and not consistent with recurrency in the brain as the effective recurrent weights tend to be relatively low (e.g. w_rec ~ 0.1), which should give you a decay. Adding this extra model experiments would help to clarify these points.

The main result of our study is that the neural network can learn to make use of existing intrinsic dynamics over the time scales used in the task. We agree with the reviewer that the space of these time scales has not been well explored and therefore we add models trained with different tau values and delays (**Supplementary Figure S8**). These also relate to points 11 and 12 below.

Learning the STD parameters is an interesting idea, but we believe it is beyond the scope of the current work. However, it should be done on tasks which are more ethologically relevant to mice and it is therefore generate an entirely new study (while detection of change is important, mice probably evolved to solve a variety of other tasks, and optimizing all the parameters, including the intrinsic time constants, might be an exaggeration).

For RNNs, we show that the learned recurrent weights are generally low in magnitude and often contain negative values (**Supplementary Figure S9**). We cannot fix the recurrent weights to too low values, or else the network will not learn.

3. Figure 2 is critical to understand the two proposed models. However, in its current form it is hard to understand and contrast the two models. For example, the full architecture could be presented in one panel highlighting that the bottom part of the model is a CNN that is pretrained on image recognition and then on top the two models further trained to do the task considered in this paper. In addition, it would be instructive to also show the output for the RNN in C-E. Also, LTP is usually used to refer to Long-term potentiation, so it would clarify things if LTSP (Long-term syn. plast.) was used instead. y-axis in D and E is missing units.

We now show the full architecture with the pretrained CNN and downstream models in panel A of Figure 2. We also make it clear that the CNN is pretrained on image recognition and only the resulting feature embeddings are used by the two downstream models. We have also added the output of example RNN units to the figure (panel E). We have also changed LTP to LTSP in the figure to avoid potential confusion and added missing units where necessary.

4. To make it possible to understand how the models are linked to the data, the models and how they were trained need to be (briefly) explained in the Results section. This should be done

before describing the model results in the "Asymmetry in the detectability" section. Also, the main text never seems to go into the detail of Fig. 2, please add this to the main text.

We have now added a brief description of how the models were trained at the beginning of the "Asymmetry in the detectability of natural images" section (**line 63**). We have also expanded on Figure 2 in the text, hopefully making it easier for the reader to understand the models used.

5. There is a lack of statistical tests throughout to support the conclusions. For example, in Fig. 3 B,C and E. But also in several other places (Fig. 6C).

We thank the reviewer for pointing this out to us. We have now performed a one-way ANOVA test, along with post-hoc Tukey's tests as a measure of statistical significance if needed. These results are now reported in the text corresponding to the figures (**"Asymmetry in the detectability of natural images", lines 78 and 94 and "Models make different predictions on image omissions", line 185**).

6. There are many important details lacking in the methods that would be essential to be able to reproduce and fully understand the work. For example the exact model used for the RNN is not given. See also the various minor points below.

We have now provided a more detailed description of the models used in the "Neural network models" section of the Methods (**line 357**). It was previously inappropriately placed in the "Neural network training" section.

Minor points

1. Title is perhaps not a good reflection of the study as adaptation can mean many different things. For example "Synaptic adaptation.." would be more accurate.

We kindly disagree with the reviewer. We propose to keep the term "adaptation" since we have not conclusively determined whether the exact mechanism is due to synaptic adaptation or intrinsic cellular mechanisms (e.g. firing rate adaptation). We have added a section in the Discussion describing this in more detail (**line 307**).

2. L21: It has been known for some time now that most synapses in the visual cortex are depressing (e.g. work by Henry Markram in the 90s and many others, see also Buchanan et al. 2012 Neuron for excitatory synapses onto other cell types).

We thank the reviewer for these additional references and have now cited them in the main text **(line 23)**.

3. This work is directly relevant to the present study and should be discussed: https://www.ncbi.nlm.nih.gov/pmc/articles/PMC6757815/

We thank the reviewer for pointing us to this additional study and have now discussed it in the "Model predictions" section of the Discussion (**line 286**). This point is also related to point 18 below.

4. Fig1: The caption should have more details. For example in B is not clear what are the two images exactly showing. Can you please also increase the resolution and size of the figures in B so that they are easier to read.

We have now provided more details in the caption of the figure, particularly for panel B. We have also increased the size and resolution of the figures in panel B for ease of viewing**.**

5. Methods: The actual equations of the firing rate approximation of the STD model should be given for clarity.

Equation 1 in the main text (Methods section) gives the firing rate approximation.

6. Methods: A L2 penalty was used to encourage sparsity. Why is this needed?

We want to clarify that the L2 penalty is used to encourage *low activations*, but not necessarily *sparsity*. **This was an error on our part, and we have now edited this in the text** (**line 419**). The use of an L2 penalty is a standard practice when training RNNs (see Masse et al., '19 and Orhan and Ma, '19). It can also be viewed as a biological constraint, where neurons are encouraged to not fire all the time.

7. Fig. 5: It would be interesting to plot the variance explained vs number of components for all the 3 cases.

We thank the reviewer for this suggestion, and have now included these plots as an additional Supplementary Figure (**Figure S4).**

8. Fig. 4: A quantitative comparison between model and data modulation indexes, and the respective summary plot would be useful here.

We thank the reviewer for this suggestion, and now report the statistical significance of the difference in change modulation indices for the models and data using a 2-sample KS test (**line 120**). There was a statistically significant difference between the experimental change modulation indices and the model change modulation indices, but that should be expected due to the fact that the models cannot fully reproduce the underlying neural mechanisms of change modulation.

9. Please clarify any pre-processing performed on the input images and if the CNN was fine-tuned on the images that were used during the (changing) task training. This is important to state since the CNN was pretrained on CIFAR-10, which is different from the input images used later on.

The CNN was only trained on the CIFAR-10 dataset and not fine-tuned on any of the images presented in the change detection task, as this could have potentially biased our results. Importantly, the images used in the experiments are not selected from the CIFAR-10 dataset. The CIFAR-10 images were first converted to grayscale and normalized to the range [0,1], followed by mean subtraction and division by the standard deviation of the dataset (mean: 0.479, std: 0.239). The same preprocessing was performed on the images used in the change detection task. We have clarified this in the "Neural network models" section of the Methods (**line 371**).

10. It would be interesting to have a high-level explanation for the importance of asymmetries in the responses to the changed images. It is not immediately obvious what the significance is of the fact that STPNet and the behavioural data show this asymmetry in the response and the RNN does not (apart from the quantitative comparison).

We have now expanded on our discussion on the importance of the observed asymmetric behavioral responses to the changed images in the Discussion section (**line 230**).

11. It would be interesting to show model experiments that vary the time between the presentation of each stimulus to compare the decay properties of the neural data vs the STPNet (this point is touched on in the "Model Predictions" section).

This is related to point 2 above and point 12 below. We have now included model experiments where we vary the length of the delay period. These results are shown in Supplementary Figure S7**.** Unfortunately, we do not have any experimental data that can be used to validate these model results, so this remains to be tested.

12. Related to the previous point and major point 2. It would be important to see how the models behave for different tau depression (STPnet) and recurrent weights (RNN).

This is related to points 2 and 11 above. We have now included model experiments where we vary the tau value. We found that the recurrent weights were generally low and could contain both positive and negative values. These results are shown in Supplementary Figures 8 and 9.

13. Its not clear exactly how many parameters the different models use, please add these to the text or ideally a table with this and the full hyperparamaters.

We now report the number of parameters in the different models as a table in the "Neural network models" section of the Methods (**Table 1**).

14. The change modulation index is not trivial to understand, adding a supp. figure that explains the index would be great.

We have now created a supplementary figure that explains the computation of the change modulation index (**Figure S1)**.

15. (in abstract): "Unlike the RNN model, STPNet also produces a similar pattern of behavior". Remove 'also'.

We thank the reviewer for pointing this out to us. We have now fixed this in the abstract.

16. Fig 2d caption: "Input-dependent changes in synaptic efficacy..." we assume that this refers to the STP model but this could be better specified.

The original panels D,E correspond to units from STPNet. We have now clarified this in the caption of Figure 2.

17. Section "Low-dimensional analyses of neural and model activity" talks about "Euclidean distance of the full population activity", but corresponding Fig. 5c caption says "The distance from the origin of the low-dimensional space is plotted using either using the Euclidean distance..". This seems to imply euclidean distance of lower-dimensional space (not full activity as implied in main text). Please clarify.

We have now clarified this in the caption of Figure 5. We used Euclidean distance of the full population activity, not in the low-dimensional space. The PC1 distance is computed with respect to the origin of the low-dimensional space.

18. In the "Model predictions" section it is mentioned that "the timescale of the short-term memory is intimately linked with the timescale of short-term depression" to solve the task. This is not necessarily a novel prediction as it is a directly follow up from other studies https://www.ncbi.nlm.nih.gov/pmc/articles/PMC6757815/. Please clarify.

We have added this reference to the “Model predictions” section discussing the relation of the current work to this prior work (**line 286**). We have also restated our claims that this is a novel prediction.

19. Methods: Clarify that the 10 random seeds correspond to 10 different model initialisations. What init. was used?

The reviewer is correct- the 10 different random seeds correspond to 10 different model initializations. We have clarified this in the text (**line 415**). The initialization used was He uniform initialization, which is the default for linear layers in Pytorch.

20. Fig. 3: Although the exact performance of the CNN is not relevant, it is relevant to show that it was trained to get reasonable accuracy on the CIFAR task to this end a standard accuracy measure should also be shown.

We have now reported the final trained accuracy of the feature preprocessing CNN we used in the Methods section (**line 372**). The models trained on CIFAR-10 achieve an average of ~63% accuracy on the test set.

Reviewer #2: The authors present two competing models for the behavior and neural activity of mice tested in a visual change detection task. One model is a recurrent neural network while the other is a network with short-term depression between the input units and the hidden layer, with not recurrent connections in the latter. The idea is to test which mechanism best captures the data and is therefore a better candidate for this particular form of short-term memory. Both networks have a small number of neurons, presumably because the task is simple to solve. The main claim of the paper is that "while both networks are able to learn the task, the STPNet model contains units whose activity are more similar to the in vivo data and produces errors which are more similar to the mice."

The authors have compared other metrics (based on dimensionality reductions, change adaptation indices in single neurons, and other quantities), and they all seem to point to the same results. They also tested their results with a network that "interpolates" between the RNN and the STPnet, i.e., it becomes one or the other net in two opposite special cases. The latter network works best, but the main signature of STP is still there and accounts for properties of the neural data that the RNN alone cannot capture.

Although this study cannot rule out the role of persistent activity (as subserved by the RNN) in short-term memory in general, their results are quite convincing that adaptation plays an important role in explaining this data. They also admit that a crucial test of their prediction would be to test the mice in a version of the task with variable inter-trial interval, since the model with STP makes clear predictions based on the time constant of recovery from depression. It is possible that both mechanisms are used, perhaps with STP being more relevant with constant (and short) intertribal intervals (as here), while persistent activity being more relevant in more difficult tasks with highly variable delay. It seems clear that, in the latter case, having a mechanism that can retain the representation of the last image would be more robust than one relying on a restricting range of adaptation processes (although adaptation on multiple and heterogeneous time scales has also been observed, and having neurons with very diverse time constants perhaps could do the job).

In conclusion, I believe the authors make a fair claim, at least in relation to this particular change detection experiment. Their methodical procedures seem appropriate to derive their conclusions.

We thank the reviewer for their positive feedback of our work. We will address their minor points below.

- Minor points:

l. 146: "(compare Figure 5G,L). These findings held over the ensemble of 146 models that were tested (Figure 5H,M)". Panels G,K and H,L instead?

We thank the reviewer for pointing this error out to us- we have now corrected this in the main text.

Reviewer #3: The authors investigate, by means of experiments and computational modeling, neuronal dynamics during a visual change detection task which requires short-term memory. They compare against experimental data, behavioral and neuronal, two models: one supporting memory storage via persistent activity (RNN), the other via short-term synaptic dynamics in the form of short-term depression, in the absence of persistent activity (STPNet). It turns out that STPNet explains better than RNN both the behavioral pattern observed (asymmetry in the detections) and the neuronal dynamics (presence of significant repeat effects in the neuronal response), to some extent.

The results presented appear correct (as far as I can say), novel to some extent, and interesting. The paper is clearly written and I found the figures well chosen and informative.

We thank the reviewer for their positive feedback on our paper and address their minor concerns below.

I have a few, essentially minor, comments.

There is a fairly large literature (experiments and modeling) on so-called match effects in non-human primates that seems, prima facie, to be relevant in the present context. For instance, the idea that some form of neuronal or synaptic “fatigue” could be responsible for repetition suppression (as observed and modeled here) is certainly not novel. I think the authors should shortly discuss the relevance (or not) of these studies for their results.

We thank the reviewer for bringing this to our attention. We have now added more discussion of this relevant literature on match effects in the “Comparison to other models” section of the Discussion (**line 268).**

Partly related to the above, Tartaglia et al. (2014) have argued that, at least in non-human primates, repetition suppression is always accompanied by match enhancement and persistent activity. At least for the match enhancement, this seems to be true also here. By looking at Fig. 4C, there is a small, but significant I would guess, fraction of neurons that have a negative change modulation index (CMI). Interestingly (if I read Fig. 4D correctly), STPNet is unable to produce negative a CMIs, while RNN produces negative (though very small compared to experiment) CMIs. Please comment on these points.

The reviewer is correct in their observation. There are some cells which show a negative change modulation index (which indicates that their activity facilitates with repetition). By design, the STPNet model cannot show negative change modulation indices due to the built-in adaptation. While in this study we focused on short-term synaptic depression, which is a dominant feature among synapses measured between cortical neurons (cite Seeman … Jarsky elife 2018), a small fraction facilitates. As such, a small fraction of cells increasing their activity could be modeled even in a feedforward network if more complex synapses are included. The RNN model has a few units that produce negative CMIs, most likely due to the fact that it does not have this constraint. We believe there is ample literature showing the role of recurrence in other forms of short-term memory. This results supports that the brain does not strictly use one

single strategy, but may employ a hybrid strategy of using adaptation, recurrence or facilitation as needed, including in this task. Our study focuses on the large importance adaptation plays in this task, but other mechanisms likely have additional contributions. We have now added some text discussing this in our paper.

Is there any persistent activity in the experimental recordings?

We did not observe any consistent patterns of persistent activity in the experimental recordings, although we are somewhat limited by the temporal resolution of the recording modality used (two-photon calcium imaging). The experimental data we analyze is from Garrett et al., 2019, who did not find persistent activity during the delay period (see their Figures 4A,B and 5A,C). Future experiments using electrophysiological recordings may be able to address this question.

Have all data underlying the figures and results presented in the manuscript been provided?

Large-scale datasets should be made available via a public repository as described in the *PLOS Computational Biology* data availability policy, and numerical data that underlies graphs or summary statistics should be provided in spreadsheet form as supporting information.

Reviewer #1: **No:** The authors state that the data is available in the metadata but I failed to find any link or pointers to this in the actual paper.

All the physiological and behavioral measurements have been described as part of the Garrett et al., 2019 ELife paper. This data is available at:
https://figshare.com/collections/Experience_shapes_activity_dynamics_and_stimulus_coding_of_VIP_inhibitory_cells/4858779/1

All the modeling results and the subsequent analysis are available at:
https://github.com/AllenInstitute/STSPNet
